# Supplementary material for: Dynamic transcriptomic profiles of zebrafish gills in response to zinc supplementation
Source: BMC Genomics. 2010 Oct 11;11:553. doi: 10.1186/1471-2164-11-553 (PMC3091702; doi:10.1186/1471-2164-11-553)
Supplement: Additional file 2 — Interactive Direct Interaction Network representing the molecular interactions between zinc, copper, iron, calcium and proteins encoded by transcripts changed by zinc supplementation. Mini web-site containing index.html and hyperlinked pages in subdirectory describing a Direct Interaction Network automatically generated based on curated interactions contained within the proprietary PathwayArchitect database. Ovals represent proteins and the circles symbolize metal ions. Objects are coloured by their abundance in zebrafish at the time-point they were significantly different from the control is a scale from -4 fold (dark green) to +4 fold (dark red). Where significant differences were found at more than one time-point, the colour overlay shows expression at the first instance. Dark blue squares denote 'binding', and light blue squares 'expression'; green squares stand for 'regulation', green diamonds for 'metabolism', and green circles for 'promoter binding'. Arrow heads indicate directionality of the interaction where annotated. All nodes and edges can be further interrogated by selecting the relative area of the image. [file 1471-2164-11-553-S2.zip › PathwayArchitect Zn xs DIN/754803.html]

# REGULATION:

|  |  |
| --- | --- |
| Type | REGULATION |
| Effect | Positive |


---

|  |  |
| --- | --- |
| Score | 0 |


---

|  |  |
| --- | --- |
| Reference Count | 22 |


---

|  |  |
| --- | --- |
| Mechanism | Unknown |


---

|  |  |
| --- | --- |
| Reference:0 || Sentence | "The hypotransferrinemic mouse (trf (hpx)) is a mutant strain exhibiting transferrin deficiency, marked anemia, hyperabsorption of iron, and elevated hepatic iron stores." |
| PMID | 10556206 |
| Year | 1999 |
| Species | Mouse |
| Journal | Blood |
| RefScore | 1 |
| Source | PArchNLP |
  |
|  |  |


---

|  |  |
| --- | --- |
 Reference:1 || Sentence | "Thus, of the three models, hpx/hpx mice showed the greatest enhancement in intestinal iron absorption and net iron-loading and provides a suitable animal model of spontaneous iron-overload." |
| PMID | 8011525 |
| Year | 1994 |
| Species | Mouse |
| Journal | Br J Haematol |
| RefScore | 1 |
| Source | PArchNLP |
  ||  |  |


---

|  |  |
| --- | --- |
 Reference:2 || Sentence | "Cellular iron and manganese uptake increases over 24 hr in cultures of normal and Hp glial cells in the presence of standard concentrations of Tf in the media; although total 59iron uptake in the Hp mouse cultures was 2X greater than normal, 54Mn uptake was similar between the two groups." |
| PMID | 9514199 |
| Year | 1998 |
| Species | Mouse |
| Journal | J Neurosci Res |
| RefScore | 2 |
| Source | PArchNLP |
  ||  |  |


---

|  |  |
| --- | --- |
 Reference:3 || Sentence | "Serum inhibitory activity has predominantly been associated with transferrin-mediated iron binding." |
| PMID | 11812214 |
| Year | 2002 |
| Species | Mouse |
|  | Human |
| Journal | Microb Pathog |
| RefScore | 0 |
| Source | PArchNLP |
  ||  |  |


---

|  |  |
| --- | --- |
 Reference:4 || Sentence | "A comparison of the lactoferrin- and the transferrin-mediated iron processing by monocytes is finally presented." |
| PMID | 3593649 |
| Year | 1987 |
| Species | Human |
|  | Mouse |
| Journal | Br J Haematol |
| RefScore | 1 |
| Source | PArchNLP |
  ||  |  |


---

|  |  |
| --- | --- |
 Reference:5 || Sentence | "The hypoxia-inducible factor-1 (HIF-1) transcriptionally up-regulates erythropoietin, transferrin, and transferrin receptor, leading to increased erythropoiesis and hematopoietic iron supply." |
| PMID | 12393745 |
| Year | 2002 |
| Species | Human |
|  | Mouse |
| Journal | Blood |
| RefScore | 1 |
| Source | PArchNLP |
  ||  |  |


---

|  |  |
| --- | --- |
 Reference:6 || Sentence | "Subsequently, iron may be linked to brain transferrin synthesized within oligodendrocytes and choroid plexus epithelial cells followed by a concomitant uptake of iron-transferrin in neurons expressing transferrin receptors." |
| PMID | 8930792 |
| Year | 1996 |
| Species | Mouse |
| Journal | J Comp Neurol |
| RefScore | 1 |
| Source | PArchNLP |
  ||  |  |


---

|  |  |
| --- | --- |
 Reference:7 || Sentence | "Transferrin (Tf), the plasma protein involved in iron transport, seems to play complex physiological roles related to cell function, differentiation and proliferation." |
| PMID | 12401962 |
| Year | 2002 |
| Species | Mouse |
|  | Human |
| Journal | Dev Neurosci |
| RefScore | 0 |
| Source | PArchNLP |
  ||  |  |


---

|  |  |
| --- | --- |
 Reference:8 || Sentence | "Atomic analysis of iron and gallium content of Sephadex G-150 fractions of treatment sera indicate that about an equimolar amount of gallium and iron are associated with transferrin." |
| PMID | 1337663 |
| Year | 1992 |
| Species | Human |
|  | Mouse |
| Journal | Am J Hematol |
| RefScore | 2 |
| Source | PArchNLP |
  ||  |  |


---

|  |  |
| --- | --- |
 Reference:9 || Sentence | "These gallium and iron concentrations result in inhibition of transferrin mediated iron uptake in vitro, and in vivo allow for > 90% saturation of transferrin with metal." |
| PMID | 1337663 |
| Year | 1992 |
| Species | Human |
|  | Mouse |
| Journal | Am J Hematol |
| RefScore | 1 |
| Source | PArchNLP |
  ||  |  |


---

|  |  |
| --- | --- |
 Reference:10 || Sentence | "Although the solute specificity of this novel transporter has not yet been determined, we speculate that it may be involved in either siderophore- or transferrin-mediated iron uptake in S. epidermidis." |
| PMID | 9673260 |
| Year | 1998 |
| Species | Mouse |
| Journal | Infect Immun |
| RefScore | 1 |
| Source | PArchNLP |
  ||  |  |


---

|  |  |
| --- | --- |
 Reference:11 || Sentence | "Two monoclonal antibodies were used which are able to block the transferrin-mediated uptake of iron by growing cells." |
| PMID | 2408900 |
| Year | 1985 |
| Species | Mouse |
| Journal | Eur J Immunol |
| RefScore | 0 |
| Source | PArchNLP |
  ||  |  |


---

|  |  |
| --- | --- |
 Reference:12 || Sentence | "The properties of monoclonal antibodies against the transferrin receptor that inhibit transferrin-mediated iron uptake are described." |
| PMID | 3545211 |
| Year | 1986 |
| Species | Mouse |
|  | Human |
| Journal | Biochem Soc Symp |
| RefScore | 0 |
| Source | PArchNLP |
  ||  |  |


---

|  |  |
| --- | --- |
 Reference:13 || Sentence | "This is associated with a profound increase in the cell surface expression of TfR, enhanced binding to fluorescein-tagged transferrin, and stimulation of transferrin-mediated iron uptake into cells." |
| PMID | 11264285 |
| Year | 2001 |
| Species | Mouse |
| Journal | J Biol Chem |
| RefScore | 1 |
| Source | PArchNLP |
  ||  |  |


---

|  |  |
| --- | --- |
 Reference:14 || Sentence | "Various types of proliferating cell are known to express transferrin receptors which are necessary for transferrin-mediated cellular iron uptake." |
| PMID | 2642388 |
| Year | 1989 |
| Species | Mouse |
| Journal | Biochim Biophys Acta |
| RefScore | 0 |
| Source | PArchNLP |
  ||  |  |


---

|  |  |
| --- | --- |
 Reference:15 || Sentence | "Methylamine also inhibited the rate of ferritin-iron incorporation, most likely by interfering with transferrin-iron release." |
| PMID | 6465862 |
| Year | 1984 |
| Species | Mouse |
| Journal | Ann Neurol |
| RefScore | 1 |
| Source | PArchNLP |
  ||  |  |


---

|  |  |
| --- | --- |
 Reference:16 || Sentence | "The similarity between the N-terminal and the C-terminal domains of MTf is much higher than that of the other transferrins, although the five amino acid residues required for iron binding were not conserved in the C-terminal domain of MTf in contrast to the conservation of these residues in both domains of the other transferrins." |
| PMID | 10542324 |
| Year | 1999 |
| Species | Mouse |
| Journal | Biochim Biophys Acta |
| RefScore | 2 |
| Source | PArchNLP |
  ||  |  |


---

|  |  |
| --- | --- |
 Reference:17 || Sentence | "This transferrin-independent iron transport coexisted with transferrin-mediated iron uptake." |
| PMID | 3948154 |
| Year | 1986 |
| Species | Mouse |
| Journal | Cancer Res |
| RefScore | 0 |
| Source | PArchNLP |
  ||  |  |


---

|  |  |
| --- | --- |
 Reference:18 || Sentence | "Iron delivery to cells is accomplished by the complexing of iron to transferrin (Tf), a monomeric iron-binding protein in the plasma, followed by specific binding of Tf to cell-surface receptors, endocytosis of the receptor-ligand complexes and ultimately, release of iron from endosomal vesicles to the cytoplasm." |
| PMID | 11062761 |
| Year | 2000 |
| Species | Mouse |
| Journal | Anticancer Res |
| RefScore | 1 |
| Source | PArchNLP |
  ||  |  |


---

|  |  |
| --- | --- |
 Reference:19 || Sentence | "Finally, when a monoclonal antibody to the mouse transferrin receptor and iron-nitriotriacetate were substituted for iron-transferrin in serum-free medium, proliferation was reduced by greater than 95%." |
| PMID | 3002966 |
| Year | 1986 |
| Species | Mouse |
|  | Human |
| Journal | Immunology |
| RefScore | 1 |
| Source | PArchNLP |
  ||  |  |


---

|  |  |
| --- | --- |
 Reference:20 || Sentence | RESULTS: Proinflammatory cytokines increase transferrin and non-transferrin bound iron uptake into human monocytes and increase transferrin-bound iron uptake by synovial fibroblasts, but have no effect on non-transferrin bound uptake into fibroblasts. |
| Year | 2004 |
| PMID | 15039637 |
| Species | Human |
| Journal | Med Sci Monit |
| RefScore | 1 |
| Source | PArchNLP |
  ||  |  |


---

|  |  |
| --- | --- |
 Reference:21 || Sentence | Labile plasma iron (LPI) represents a component of non-transferrin-bound iron (NTBI) that is both redox-active and chelatable, capable of permeating into organs and inducing tissue iron overload. |
| PMID | 15737890 |
| Year | 2005 |
| Species | Human |
| Journal | Best Pract Res Clin Haematol |
| RefScore | 1 |
| Source | PArchNLP |
  |


---

|  |  |
| --- | --- |
